# Metagenomic next-generation sequencing as a diagnostic tool for toxoplasmic encephalitis

**DOI:** 10.1186/s12941-018-0298-1

**Published:** 2018-12-26

**Authors:** Zhiliang Hu, Xing Weng, Chunhua Xu, Yang Lin, Cong Cheng, Hongxia Wei, Wei Chen

**Affiliations:** 1 Department of Infectious Disease, the Second Hospital of Nanjing, Nanjing University of Chinese Medicine, 1-1 Zhongfu Road, Nanjing, 210003 China; 2 Center for Global Health, School of Public Health, Nanjing Medical University, Nanjing, 211166 China; 3 Department of Pathogen Detection Products, BGI-Shenzhen, Shenzhen, 518083 China; 4 Department of Research and Development of Infectious Diseases, BGI-Wuhan, Wuhan, 430075 China; 5 Department of Clinical Research Center, the Second Hospital of Nanjing, Nanjing University of Chinese Medicine, 1-1 Zhongfu Road, Nanjing, 210003 China

**Keywords:** Next-generation sequencing, Toxoplasmic encephalitis, Diffuse brain lesions, *Toxoplasma gondii*, Human immunodeficiency virus

## Abstract

**Background:**

More than 100 different pathogens can cause encephalitis. Testing of all the neurological pathogens by conventional methods can be difficult. Metagenomic next-generation sequencing (NGS) could identify the infectious agents in a target-independent manner. The role of this novel method in clinical diagnostic microbiology still needs to be evaluated. In present study, we used metagenomic NGS to search for an infectious etiology in a human immunodeficiency virus (HIV)-infected patient with lethally diffuse brain lesions. Sequences mapping to *Toxoplasma gondii* were unexpectedly detected.

**Case presentation:**

A 31-year-old HIV-infected patient presented to hospital in a critical ill condition with a Glasgow coma scale score of 3. Brain magnetic resonance imaging showed diffuse brain abnormalities with contrast enhancement. Metagenomic NGS was performed on DNA extract from 300 μL patient’s cerebrospinal fluid (CSF) with the BGISEQ-50 platform. The sequencing detection identified 65,357 sequence reads uniquely aligned to the *Toxoplasma gondii* genome. Presence of *Toxoplasma gondii* genome in CSF was further verified by *Toxoplasma gondii*-specific polymerase chain reaction and Sanger sequencing. Altogether, those results confirmed the diagnosis of toxoplasmic encephalitis.

**Conclusions:**

This study suggests that metagenomic NGS may be a useful diagnostic tool for toxoplasmic encephalitis. As metagenomic NGS is able to identify all pathogens in a single run, it may be a promising strategy to explore the clinical causative pathogens in central nervous system infections with atypical features.

## Background

More than 100 different pathogens can cause encephalitis [1]. Testing of all the neurological pathogens by conventional methods, such as smear, serologic tests, culture, and pathogen-specific polymerase chain reaction (PCR), can be difficult. Metagenomic next-generation sequencing (NGS) has the advantage of identification and genomic characterization of infectious agents in a target-independent manner [2]. Accumulating evidences have shown that metagenomic NGS may help to identify the etiological agent when conventional methods have failed [3]. As this strategy do not require a priori knowledge of a specific pathogen, it may be especially useful for diagnosis of case with atypical presentation. In the present study, we used metagenomic NGS to search for an infectious etiology in a human immunodeficiency virus (HIV)-infected patient with lethally diffuse brain lesions. Sequences mapping to *Toxoplasma gondii* were unexpectedly detected by NGS of the cerebrospinal fluid (CSF) sample.

## Case presentation

**Fig. 1:**
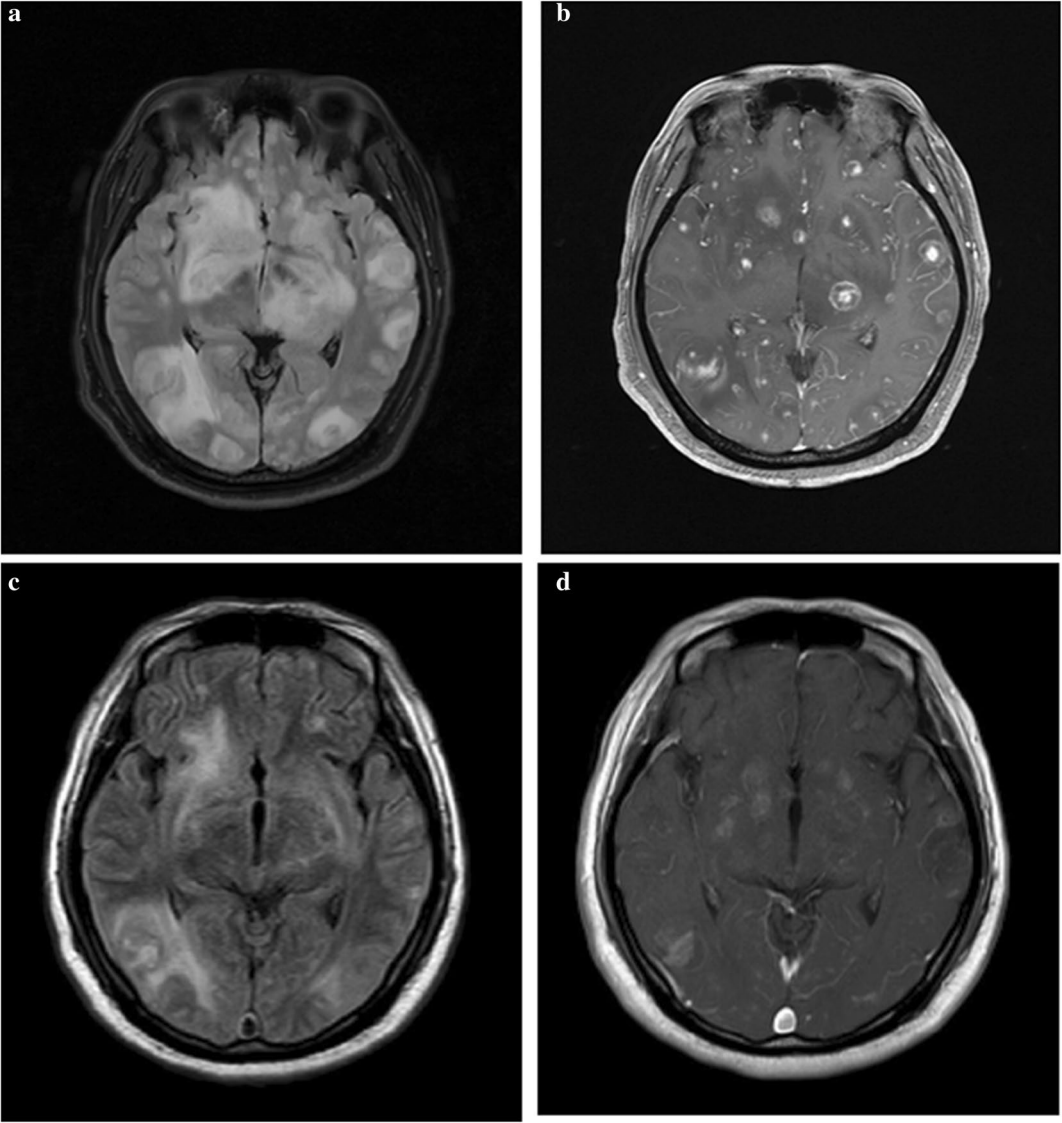
Brain magnetic resonance imaging before and after anti-*Toxoplasma gondii* therapy. At admission, brain magnetic resonance imaging (MRI) showed multiple brain abnormalities (**a** T2-FLARE imaging) with enhancement (**b** contrast-enhanced MRI). After treated as toxoplasmosis encephalitis for 11 days, a following-up brain MRI showed significant improvement of brain lesions(**c** T2-FLARE imaging; **d** contrast-enhanced MRI)

A 31-year-old HIV-infected patient with a CD4 cell count of 2 cells/μL was admitted to our hospital because of fever and headache for 20 days and disturbance of consciousness for 7 days. At admission, he had a Glasgow coma scale score of 3. A brain magnetic resonance imaging (MRI) showed diffuse brain abnormalities with contrast enhancement (Fig. 1a, b). He was empirically treated as toxoplasmosis encephalitis (TE) with azithromycin (0.5 g everyday intravenously) plus co-trimoxazole (1.44 g every 8 h through nasogastric tube) according to local guidelines [4]. Also, a combinatory therapy with isoniazid, rifampicin, ethambutol, pyrazinamide, linezolid and cefotaxime-sulbactam was administered to ensure the coverage of *Mycobacterium* tuberculosis and common bacteria.

After his admission, tests from the blood samples showed no sign of tuberculosis (negative interferon-gamma release assay; negative *Mycobacterium* tuberculosis antibody) and fungal infection (negative 1-3-β-d-glucan and galactomannan assay). Serum anti-toxoplasma antibody immunoglobulin G (IgG) was positive, while anti-toxoplasma antibody immunoglobulin M (IgM) was negative. A lumbar puncture showed increased intracranial pressure (250 mmH_2_O). CSF analysis demonstrated a white blood cell count of 7 cells/mm^3^ and normal protein and glucose levels. India ink staining and acid-fast staining of CSF were negative. CSF treponema pallidum particle agglutination assay, cryptococcal antigen, culture, and Gene Xpert MTB/RIF assay were also negative. An unbiased metagenomic sequencing of CSF revealed high burden of *Toxoplasma gondii*. Anti-tuberculosis drugs were subsequently discontinued. After treated as toxoplasmosis encephalitis for 11 days, the patient’s Glasgow coma scale score increased to 9. A brain MRI demonstrated significant improvement of brain lesions (Fig. 1c, d). He then developed rash and had high fever again which was thought to be an allergic reaction to co-trimoxazole. For complex reasons, his family gave up therapy and the patient was lost to follow-up.

### Metagenomic NGS

**Fig. 2:**
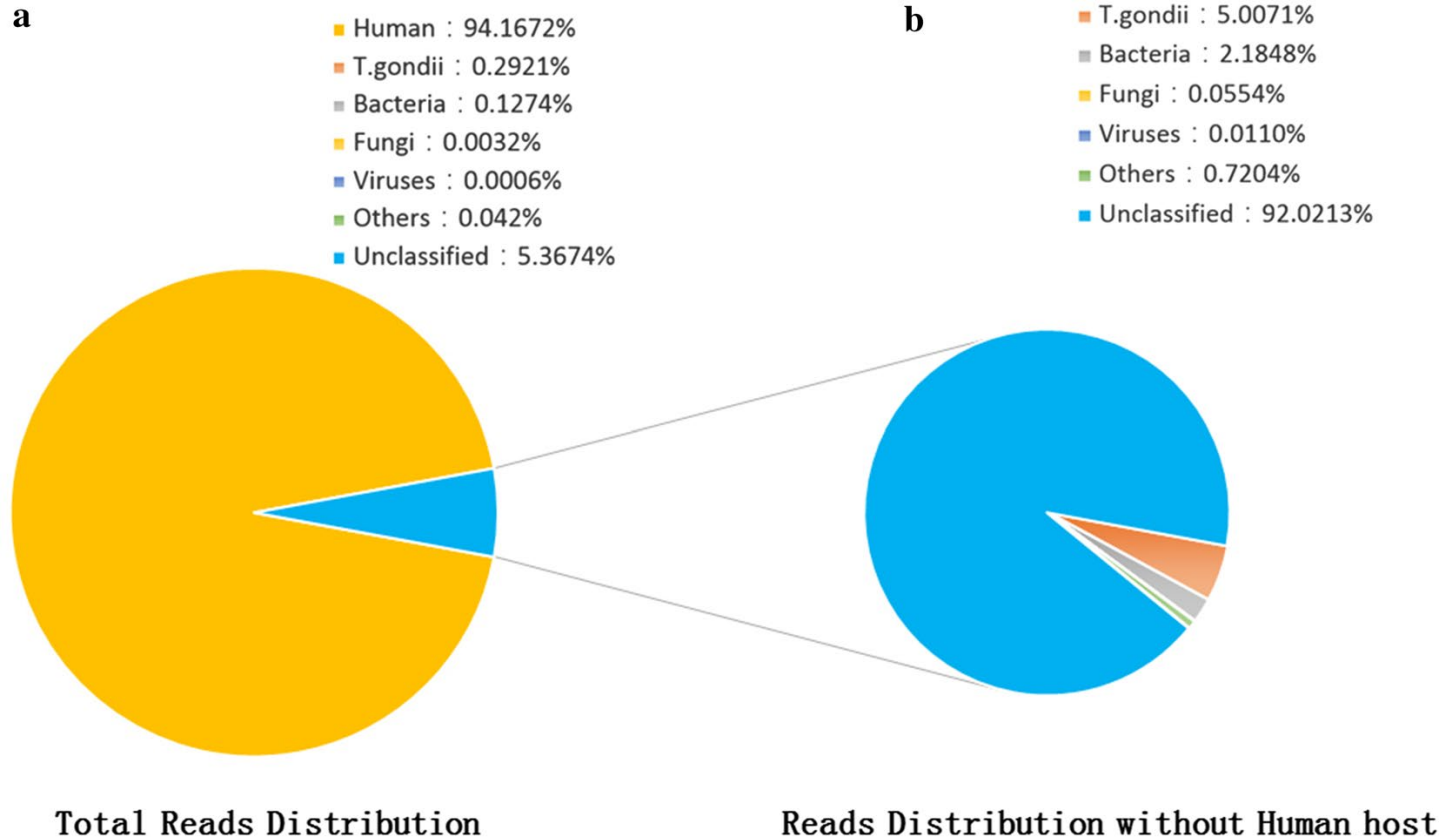
Analysis of sequencing result of *T.gondii* using the NGS method. **a** Reads distribution of total DNA in the CSF samples; **b** reads distribution of all non-human reads

The patient’s CSF sample was sent for unbiased pathogen detection by metagenomic NGS at BGI-Shenzhen. DNA was extracted from 300 μL CSF sample using the TIANamp Micro DNA Kit (DP316, TIANGEN BIOTECH) according to the manufacturer’s recommendation and sonicated to a size of 200–300 bps fragments (Bioruptor Pico protocols). Then, DNA libraries were constructed via end-repaired adaptation and PCR amplification. The quality of the DNA libraries was evaluated using an Agilent 2100 Bioanalyzer (Agilent Technologies, Santa Clara, CA) combined with quantitative PCR to measure the adapters. Qualified libraries were sequenced by BGISEQ-50 platform (BGI-Tianjin, Tianjin, China) [5]. With screening of human host sequences via Burrows- Wheeler Alignment tool [6], high-quality sequencing data were aligned with Microbial Genome Databases, which is composed of 2700 whole genome sequences of viral taxa, 1494 bacterial genomes or scaffolds, 73 fungi and 47 parasites related with human infectivity. The total number of reads from different samples was standardized as 20 M for comparison. The mapped data were further processed and with the depth and coverage of each species calculated using Soap Coverage (http://soap.genomics.org.cn). A control sample from a non-infected patient was obtained and subjected to the aforementioned procedures. The sequencing detection identified 65,357 (out of 22,378,551) sequence reads (0.29%) uniquely aligned to the *Toxoplasma gondii* genome (Fig. 2a), and these reads covered a high percentage (13.02%) of the *Toxoplasma gondii* genome. When the reads from the human host were excluded, *Toxoplasma gondii* reads showed dominant abundance in all microbial species, accounting for 5.01% of total non-human reads (Fig. 2b). No reads of *Toxoplasma gondii* were detected from control samples.

### *Toxoplasma gondii*-specific PCR and Sanger sequencing

**Fig. 3:**
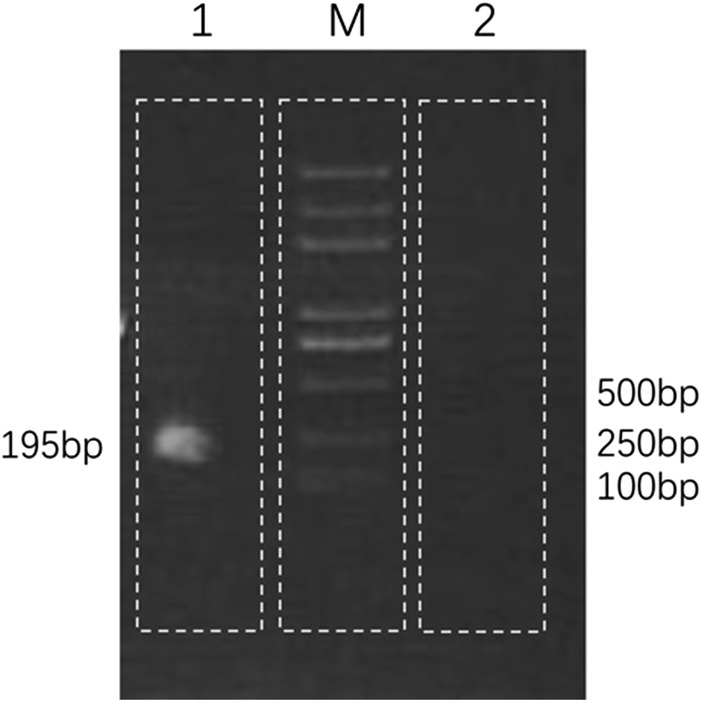
PCR detection of *Toxoplasma gondii*. Lane 1: 195-bp PCR product of the B1 gene of *Toxoplasma gondii*; Lane M: DNA ladder. Lane 2: Negative control

Sequence-specific PCR was conducted to amplify the glycerol-3-phosphate dehydrogenase (B1) gene of *Toxoplasma gondii* with specific primers TOXO 1 (GGAACTGCATCCGTTCATGAG) and TOXO 2 (TCTTTAAAGCGTTCGTGGTC) for verification of the results of NGS [7]. The PCR products (Fig. 3) were sequenced using an ABI PRISM 3730 DNA Sequencer (Applied Biosystems, Foster City, CA, USA). The sequences were then mapped to the nucleotide database with the online NCBI blast. A 195 bp consensus sequence of the B1 gene of *Toxoplasma gondii* was assembled and found to be 100% identical to a reference *Toxoplasma gondii* sequence (GenBank accession no. KX270373). Consequently, all these results indicated that the patient was infected with *T. gondii*.

## Discussion

TE is one of the most important neurological opportunistic infections in HIV-infected patients with a CD4 T cell count less than 100 cells/μl [8]. The pathogen causing TE is *Toxoplasma gondii* which is difficult to culture and is insensitively detected by microscopic examination of CSF [9]. The serum anti-toxoplasma IgM antibody in HIV-infected patients with TE is usually negative; although serum anti-toxoplasma IgG antibody is almost uniformly positive, it could not be used to differentiate between latent infection and reactivation [10]. Currently, the most common method capable of etiologically confirming TE is the CSF *Toxoplasma gondii* PCR with high specificity but variable sensitivities (35–72%) in different PCR systems [11]. However, commercial PCR kit is still unavailable in many regions including China and such assays typically require an a priori suspicion of *T. gondii* infection.

For the above mentioned reasons, the diagnosis of TE in HIV-infected patients is still challenging. Most cases of the TE have been diagnosed empirically relying on clinical and radiographic responses to anti-*Toxoplasma gondii* therapy without possible alternative explanation [10]. Even in recently published article, those criteria have been considered “gold standard” for diagnosing TE [12]. This diagnostic strategy, although widely used in clinical centers including our center, is not optimal for managing HIV-infected patients with brain lesions, which could be related to multiple etiologies including but not limited to TE, central nervous system (CNS) tuberculosis or cryptococcosis, as well as primary CNS lymphoma [8, 13, 14]. The brain imaging features are not specific enough to make a definite diagnosis. Here, in this study, using a novel metagenomic NGS platform, we were able to detecting *Toxoplasma gondii* in CSF sample and finally confirmed the etiology of the brain lesions. The results indicated that metagenomic NGS may be useful diagnostic tool for TE in HIV-infected patients.

To establish a diagnosis of CNS infection, traditional methods, such as smear, culture, nucleic acid amplification assays and serological tests, generally require collecting massive volume of CSF sample to perform a battery of tests. However, metagenomic NGS has the capability to identify all pathogens directly from CSF sample with a single run in a hypothesis-free and culture-independent manner. This method which allows for universal pathogen detection could be applied for discovering novel organisms [2]. With regard to our patient, diffuse brain contrast-enhancing lesions were not typical imaging features of common HIV-associated opportunistic CNS infections [8]. A consensus for initial clinical diagnosis of the brain lesions was not reached. In this condition, we used metagenomic NGS of CSF sample to explore pathogens that were not efficiently detected by traditional methods, as well as to discovery possible novel pathogens. Of note, the studied patient was in a critical condition. Empiric broad-spectrum therapy with multiple antimicrobials was administered to ensure maximum coverage of possible common CNS pathogens. The turn-around time for NGS platform used in our study is about 30–35 h from specimen receipt. With this platform, a timely etiologic diagnosis may be achieved, which could help to narrow antimicrobial therapy therefore reduce cost and drug-related toxicities.

Despite raising publication of metagenomic NGS successes, limitation of this technique needs to be noted, including contamination of nucleic acid from reaction kit, differentiation of colonization from infection, laboratory procedures and bioinformatic analysis standardization and clinical interpretation. With increasing application of metagenomic NGS assays, it is significant for clinicians to fully understand both the benefits and limitations of this assay for diagnosis of infection [15]. In our study, except *T. gondii*, microbes identified in the infected patient but not in the control were considered as background introduced during procedure of sample collection. These microbes showed relative low number of reads, and have been reported to be environmental microorganisms. Nevertheless, metagenomic NGS could not, by itself, define the source of the detected microbial DNA. In the case of suspected environmental microbial contamination, a multidisciplinary discussion by clinicians, microbiologists as well as the lab technicians would help to interpret the results.

An interesting question regarding the metagenomic NGS is whether this technology is more sensitive than traditional methods for pathogen detection? Metagenomic NGS has been shown to be more sensitive than traditional bacterial culture method for diagnosing bacteremia [16]. In a recent study by Guo et al. when metagenomic NGS was used, the sensitivity of bacterial meningitis diagnosing increased from 55.6 to 68.7% [17]. All those studies suggest that metagenomic NGS is more sensitive than traditional culture method. However, it is less clear whether metagenomic NGS has higher sensitivity compared with other molecular technologies (such as pathogen-specific PCR) for etiologically diagnosis of infectious diseases.

As shown in our study, the vast majority of the DNA sequences detected by metagenomic NGS were from human genome DNA. Presence of massive human DNA would influence the depth of sequencing of the causative microorganism. To overcome this limitation, future study may focus on properly preliminary treatment of sample that could maximumly remove most of human DNA while do not impede recovery of pathogen DNA. At last, the approach needs a lot of bioinformatic power to analyze one sample that this approach is relatively expensive compared with conventional methods for pathogen detection. In our opinion, bioinformatic power would not be a bottle-neck in next 10–20 years, as technologies of metagenomic NGS and computer science are continuingly being improved. Different bioinformatic powers could be designed to customize for different purposes of analysis.

## Conclusions

In the current report, TE with atypical brain imaging features in an HIV-infected patient was rapidly diagnosed using unbiased metagenomic NGS. This study suggests that metagenomic NGS may be a useful diagnostic tool for TE. More importantly, as metagenomic NGS is able to identify all pathogens in a single run, it may be a promising strategy to explore the clinically causative pathogens in CNS infections, especially when the clinical features are atypical.

## Abbreviations

PCR: polymerase chain reaction
NGS: next-generation sequencing
HIV: human immunodeficiency virus
CSF: cerebrospinal fluid
MRI: magnetic resonance imaging
TE: toxoplasmosis encephalitis
IgG: immunoglobulin G
IgM: immunoglobulin M
CNS: central nervous system
